# Peripheral Lymphocytes of Patients with Inflammatory Bowel Disease Have Altered Concentrations of Key Apoptosis Players: Preliminary Results

**DOI:** 10.1155/2018/4961753

**Published:** 2018-11-01

**Authors:** Katarzyna Neubauer, Barbara Woźniak-Stolarska, Małgorzata Krzystek-Korpacka

**Affiliations:** ^1^Department of Gastroenterology and Hepatology, Wroclaw Medical University, 50-556 Wroclaw, Poland; ^2^Department of Medical Biochemistry, Wroclaw Medical University, 50-368 Wroclaw, Poland

## Abstract

Notwithstanding uncertain pathogenesis of inflammatory bowel disease (IBD), deregulation of adaptive immunity is paramount for the development of inflammation. Essential role in the resolution of inflammation is played by apoptosis, deregulated in lymphocytes isolated from inflamed intestine. Despite IBD being a systemic disease, little is known about apoptosis of peripheral lymphocytes. The concentrations of Bcl-2, cytochrome c, p53, and caspase-9 were determined (ELISA) in lymphocyte-enriched fractions of peripheral blood mononuclear cells (LE-PBMCs) from 64 individuals (42 with IBD) and related to IBD phenotype and activity, treatment, and inflammatory and hematological indices. The diagnostic potential of evaluated markers was determined as well. All evaluated molecules were significantly lower in IBD patients, of which cytochrome c and p53 were significantly lower exclusively in patients with Crohn's disease (CD) and cytochrome c differed significantly between CD and ulcerative colitis (UC). Caspase 9 was significantly lower in active IBD and Bcl-2 in active UC whereas cytochrome c was higher in active CD. Treatment with corticosteroids affected the concentrations of cytochrome c and p53. Both positively correlated with hsCRP and the concentrations of all markers were interrelated. As IBD markers, Bcl-2 and caspase-9 displayed good accuracy and, as a panel of markers with cytochrome c, their accuracy was excellent (92%). As CD markers Bcl-2, cytochrome c, and p53 displayed fair accuracy but combined determination of Bcl-2 and cytochrome c improved the accuracy to 85%. Taken together, our results imply diminished intrinsic apoptotic capacity of LE-PBMCs in IBD but an upregulation of proapoptotic features parallel to increasing severity of inflammation. Observed abnormalities in intrinsic pathway of apoptosis are more pronounced in CD. Upon positive validation on a larger set of patients, combined quantification of Bcl-2 and cytochrome c might be considered as an adjunct in differential diagnosis of UC and CD of colon and rectum.

## 1. Introduction

Inflammatory bowel disease (IBD) is chronic, incurable conditions of digestive tract decreasing patients' quality of life and affecting currently more than five million people all over the world [1]. Two major forms of the disease are Crohn's disease (CD) and ulcerative colitis (UC). Cardinal features of IBD are increasing incidence worldwide, complex diagnostic process, relapsing-remitting pattern, and unclear multifactorial pathogenesis. Compound background of the development of IBD involves interplay between immune system (the* immunome*) and the gut microbiota (the* microbiome*) in individuals with genetic predisposition (the* genome*) in presence of the environmental factors (the* exposome*) [2]. As a consequence there is no causative treatment available and administrated therapeutic strategies continue to depend mostly on systemic immunosuppression being just one link in the complicated chain of interactions. Introduction of biologic therapy directed against tumor necrosis factor (TNF)-*α* over two decades ago improved clinical outcomes in IBD patients and paved the way for other immunotherapies [3]; still the effectiveness of IBD treatment does not exceed 50% [4]. With new therapeutic modalities being tested and implemented to the clinical practice, noninvasive biomarkers, which may assist in the diagnosing and monitoring of IBD as well as in predicting the disease course and treatment effectiveness, are needed [5].

Despite uncertain pathogenesis of the disease, deregulation of the mechanisms of adaptive immunity, including excessive T-cell responses towards commensal and/or pathogenic gut microbiota as well as lymphocyte resistance to desensitization signals, is paramount for, respectively, the initiation and perpetuation of inflammatory response in IBD [6]. Essential role in the resolution of inflammation,* via* the regulation of various T cell populations, is played by apoptosis. It is a physiological process of programmed cell death, responsible for the elimination of unnecessary, aged, or damaged cells [7]. Apoptosis is initiated by the activation of death-receptors at the plasma membrane by ligands such as TNF-*α* or Fas (extrinsic pathway) or by the release of mitochondrial constituents such as cytochrome c (intrinsic pathway). Disrupted apoptosis may lead to the development of a number of pathologies including autoimmune diseases and malignancies. As such, the process is strictly regulated and controlled. Among others, transcription factor p53 and the members of Bcl-2 family, consisting of both pro- and antiapoptotic mediators, play a critical role in the regulation of intrinsic pathway of programmed cell death [8].

Characteristic feature of an inflamed mucosa from IBD patients is its infiltration with lymphocytes T [9]. An increased level of antiapoptotic mediators as well as an aberrant response to proapoptotic signals has been previously observed in lymphocytes isolated from intestinal lamina propria of IBD patients [10–13]. Discrepancies exist concerning whether the abnormalities in mucosal lymphocyte apoptosis concern both UC and CD or CD alone. Nevertheless, enhanced survival and resistance to apoptosis of infiltrating T cells have been implicated in the disease pathogenesis and/or exacerbation [7]. Moreover, inducing lymphocyte apoptosis is one of the modes of action of anti-TNF*α* agents such as infliximab and adalimumab [14]. It is also an aim of prospective immunotherapies, such as extracorporeal photopheresis (ECP) in which patients' lymphocytes are exposed to a photoactivatable drug with cytotoxic effect. ECP has recently been successfully tested in CD patients with the disease refractory to immunosuppression and/or anti-TNF therapies (reviewed in [15]).

Being accompanied by a wide spectrum of extraintestinal manifestations IBD gains systemic character and is no longer considered the disease of exclusively gastrointestinal tract [16]. Yet, previous studies have mainly focused on apoptosis resistance of mucosal T cells whereas little is known about the susceptibility to apoptosis of peripheral lymphocytes. Thus, we performed this pilot study to evaluate the concentrations of proapoptotic p53, cytochrome c, and caspase 9 and the level of antiapoptotic Bcl-2 in healthy volunteers and IBD patients with respect to the disease type, activity, and treatment. Additionally, we evaluated these apoptosis modulators as potential biomarkers in IBD.

## 2. Materials and methods

### 2.1. Patients

A group of 64 individuals was enrolled for current study: 42 IBD patients (25 with ulcerative colitis and 17 with Crohn's disease) from the Department of Gastroenterology and Hepatology, Wroclaw Medical University, and 22 apparently healthy volunteers recruited from among the hospital staff.

Exclusion criteria for IBD patients were as follows: indeterminate colitis, coexistence of other severe systemic diseases, cancer disease, liver diseases, or pregnancies. The Crohn's Disease Activity Index (CDAI), combining the evaluation of vital parameters, clinical findings, and medical history (as described in detail elsewhere [17]), was applied for the assessment of CD activity and the Mayo Scoring System (MDAI), encompassing the evaluation of stool frequency and rectal bleeding, endoscopic findings, and the physician's global assessment [18], was applied for the evaluation of UC activity. Additionally, Truelove and Witts criteria were applied to express the disease severity in UC patients. The criteria are based on number of bloody stools per day, temperature, heart rate, erythrocyte sedimentation rate (ESR), and hemoglobin concentration, categorizing UC as mild, moderate, or severe [19]. Detailed demographic and clinical data on study participants, together with treatment with azathioprine (AZA), corticosteroids (CS), and 5′-aminosalicilic acid (ASA), are given in Table 1.

The study protocol was accepted by the Medical Ethics Committees of Wroclaw Medical University. The study was conducted in accordance with the Helsinki Declaration of 1975, as revised in 1983, and an informed consent was obtained from all study participants.

### 2.2. Analytical Methods

Whole blood was drawn by venipuncture in a fasting state. Peripheral blood mononuclear cells (PBMCs) were isolated from whole blood by density gradient centrifugation (400 g, 20°C, 30 minutes) using Ficoll-Paque PLUS (1,077 g/ml) (GE Healthcare, Little Chalfont, UK), washed three times with phosphate buffer saline (5:1) and centrifuged (200 g, 20°C, 10 minutes). Subsequently, PBMCs were subjected to the density gradient centrifugation (400 g, 20°C, 20 minutes) using Percoll (1.131 g/ml; GE Healthcare), followed by the washing of collected lymphocyte fraction of PBMCs with phosphate buffer saline. The applied method allows for obtaining lymphocytes with purity up to 99% [20]. Obtained cells are further referred to as lymphocyte-enriched PBMCs (LE-PBMCs).

Cytochrome c was measured immunoenzymatically in the supernatant of cell lysate using Cytochrome C Human ELISA assay according to manufacturer's instructions (Bender MedSystems GmbH, Vienna, Austria). For cytochrome c determination, 1.5×10^6^ LE-PBMCs were resuspended in lysis buffer provided with an assay and allowing for selective disruption of plasma membrane and the quantification of cytoplasmic fraction of cytochrome c, as declared by the manufacturer. For Bcl-2, p53, and caspase 9 determination, 5×10^6^ LE-PBMCs were resuspended in lysis buffers and quantified immunoenzymatically in the supernatants of cell lysates using, respectively, Human Bcl-2 Platinum ELISA, Human p53 Platinum ELISA kits, and Human Caspase-9 Platinum ELISA from the same manufacturer following attached protocols.

Cells were counted with Countess(R) Automated Cell Counter (Life Technologies, CA, USA) and the results presented are expressed per cell number (1.5×10^6^ LE-PBMCs in case of cytochrome c and 5×10^6^ LE-PBMCs in case of other compounds). Additionally, the obtained results were verified using protein concentration in cell lysates as a normalization factor. Protein concentration was determined using Bradford method and Bio-Rad Protein Assay with bovine serum albumin as a standard (BioRad, CA, USA).

### 2.3. Statistical Analysis

Data distribution was tested using* Chi*-square test and homogeneity of variances using Levene's test. Data were log-transformed to obtain normality of distribution and/or homogeneity of variances. Normally distributed data were analyzed using one-way ANOVA with Student-Newman-Keuls* post hoc* test (multigroup comparisons) and* t*-test for independent samples (two-group comparisons). Non-normally distributed data were analyzed using Kruskal-Wallis* H* test Conover with* post hoc* test (multigroup comparisons) and Mann-Whitney* U* test (two-group comparisons). Data are presented as arithmetic means (normally distributed data), geometric means (log-transformed data), or medians (nonnormally distributed data) and accompanied with 95% confidence interval (*CI*) [21]. Analysis of covariance (ANCOVA) was used to account for potential variation in cell number (expressed as variation in protein concentration between cell lysates) and to discern variables independently associated with examined mediators of apoptosis. Data were log-transformed prior to ANCOVA. Correlation analysis was conducted using Pearson's or Spearman's rank correlation tests. Frequency analysis was conducted using the Chi-square test. The relevance of examined regulators of apoptosis as indicators of IBD in general or CD alone was tested using the Receiver Operating Characteristics (ROC) curve analysis. The overall performance was expressed as the area under the ROC curve (AUC). Additionally, an optimal cut-off value with accompanying sensitivities and specificities was determined. Also, logistic regression was used to calculate discriminative power of combined markers. Sample size analysis was conducted for comparison of means and AUCs to determine the number of observations for the analyses to be reasonably powered.

All calculated* p* values were two-sided, Bonferroni correction was used in case of multiple testing, and p≤0.05 was considered statistically significant. Statistical analysis was conducted with MedCalc Statistical Software version 17.6 (MedCalc Software bvba, Ostend, Belgium; http://www.medcalc.org; 2017).

## 3. Results

### 3.1. Bcl-2 in IBD

**Figure 1:**
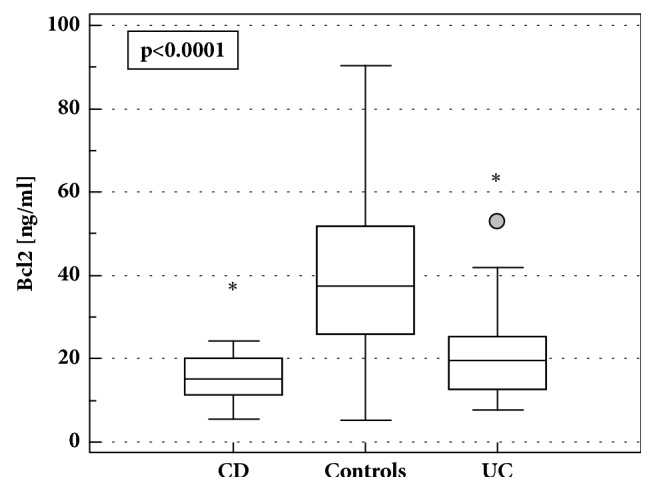
Bcl-2 concentrations in LE-PBMCs lysates from IBD patients and healthy controls. Data presented as medians with 95%CI and analyzed using Kruskal-Wallis* H* test with Conover* post hoc* test. Boxes represent interquartile range, horizontal line – median value, whiskers - 95% CI, and grey dot – far-out values. *∗*, statistically different from controls; CD, Crohn's disease; UC, ulcerative colitis.

The concentrations of Bcl-2 in LE-PBMCs from IBD patients were significantly lower as compared to healthy volunteers (16.8 ng/ml (14.5-19.5)* vs*. 35.1 ng/ml (26.3-46.7), p<0.001). Bcl-2 was lower both in CD and in UC (Figure 1) without significant difference between both conditions (*p*=0.063). The relation remained significant following adjustment to protein concentration in cell lysates (*p*<0.001). Since there were significant differences in AZA treatment between CD and UC patients, data were reanalyzed using ANCOVA. Also with AZA treatment and differences in protein concentrations accounted for, there was only tendency towards higher concentrations of Bcl-2 in UC patients (p=0.075).

There was no difference in median Bcl-2 concentrations in LE-PBMCs from CD patients with active and inactive CD (15 ng/ml (9.9-20.9)* vs*. 14.6 ng/ml (12.3-18),* p*=0.956) and no significant correlation with CDAI (*p*=0.374). Patients with active UC, however, had significantly lower Bcl-2 as compared to patients with inactive UC (18.2 ng/ml (14.1-22.3)* vs*. 27.5 ng/ml (15.8-39.2),* p*=0.044), although there was no significant correlation with MDAI (*p*=0.464). The difference between patients with active and inactive UC was more pronounced when CS treatment and differences in protein concentrations were accounted for (*p*=0.027).

### 3.2. Cytochrome c in IBD

**Figure 2:**
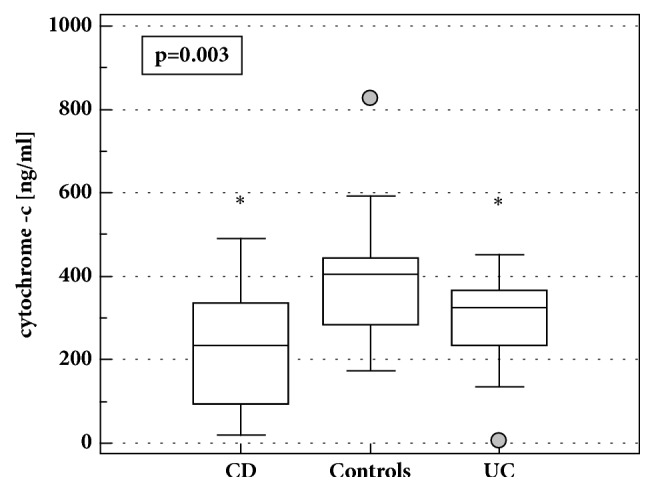
Cytochrome c concentrations in LE-PBMCs lysates from IBD patients and healthy controls. Data presented as medians with 95%CI and analyzed using Kruskal-Wallis* H* test with Conover* post hoc* test. Boxes represent interquartile range, horizontal line – median value, whiskers - 95% CI, and grey dot – far-out values. *∗*, statistically different from controls; CD, Crohn's disease; UC, ulcerative colitis.

The concentrations of cytochrome c in LE-PBMCs from IBD patients were significantly lower as compared to healthy volunteers (213 ng/ml (169-282)* vs*. 363 ng/ml (309-426),* p*=0.002). When analyzed separately, both CD and UC patients had significantly lower cytochrome c (Figure 2). The relation remained significant following adjustment to protein concentration in cell lysates (*p*=0.012). There was no significant difference between CD and UC. However, after accounting for differences in protein concentration, the disease activity, and AZA treatment, patients with CD had significantly lower cytochrome c levels than UC patients (*p*=0.024).

On average, patients with active CD have significantly higher cytochrome c levels than these with inactive disease (213 ng/ml (145-313)* vs*. 75.5 ng/ml (10-562),* p*=0.039) but there was no significant correlation between cytochrome c concentrations and CDAI (*p*=0.506). ANOCOVA with CS treatment and protein concentration accounted for showed that in CD patients both the disease activity (*p*=0.012) and treatment (*p*=0.010) had significant impact on cytochrome c concentrations in LE-PBMCs. Exclusively in CD patients, cytochrome c concentrations correlated positively with PLT count (*r*=0.52,* p*=0.031) and negatively with HGB concentrations (*r*= -0.52,* p*=0.032).

There was no significant difference between patients with active and inactive UC (295 ng/ml (234-356)* vs*. 307 ng/ml (237-377),* p*=0.807). There was no significant correlation with MDAI (*p*=0.416). However, cytochrome c concentrations tended (*p*=0.070) to increase along with increased Truelove and Witts severity index with 244±119, 339±88, and 349±37 ng/ml in mild, moderate, and severe UC, respectively. They were also positively correlated with hsCRP concentrations (*r*=0.42,* p*=0.039).

### 3.3. p53 in IBD

**Figure 3:**
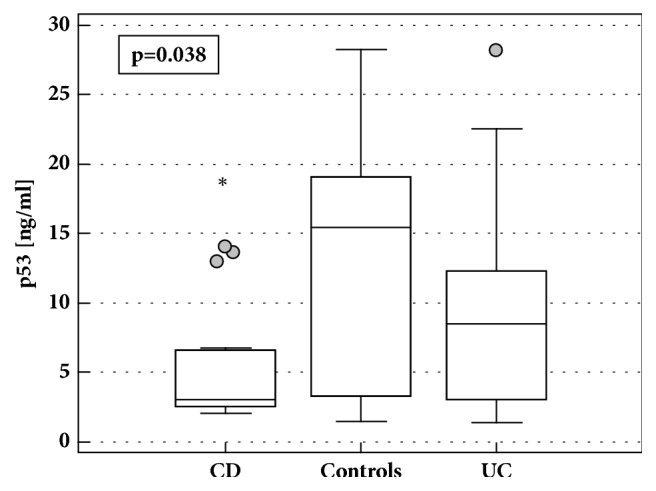
p53 concentrations in LE-PBMCs lysates from IBD patients and healthy controls. Data presented as medians with 95%*CI* and analyzed using Kruskal-Wallis* H* test with Conover* post hoc* test. Boxes represent interquartile range, horizontal line – median value, whiskers - 95% CI, and grey dot – far-out values. *∗*, statistically different from controls; CD, Crohn's disease; UC, ulcerative colitis.

The concentrations of p53 in PBLs from IBD patients tended to be lower than in healthy volunteers but the difference did not reach statistical significance (4.83 ng/ml (3.04-9)* vs*. 15.5 ng/ml (4.58-18.43),* p*=0.080). However, when analyzed separately, CD patients had significantly lower p53 than healthy individuals (Figure 3), also when adjusted to the differences in protein concentrations between cell lysates (*p*=0.029).There was significant difference between CD and UC patients following adjustment to the differences in AZA treatment and protein concentrations (*p*=0.028).

There was no difference in median p53 concentrations in LE-PBMCs from CD patients with active and inactive CD (3.2 ng/ml (2.5-9.7)* vs*. 2.8 ng/ml (2.5-4.7),* p*=0.477) and no significant correlation with CDAI (*p*=0.948). Also, there was no difference in patients with active and inactive UC (6.1 ng/ml (3.8-9.7)* vs.* 8.6 ng/ml (4.9-15),* p*=0.351) and p53 concentrations did not correlate with MDAI (*p*=0.262).

The concentrations of p53 in LE-PBMCs of UC patients correlated positively with hsCRP (*ρ*=0.42,* p*=0.040).

### 3.4. Caspase 9

**Figure 4:**
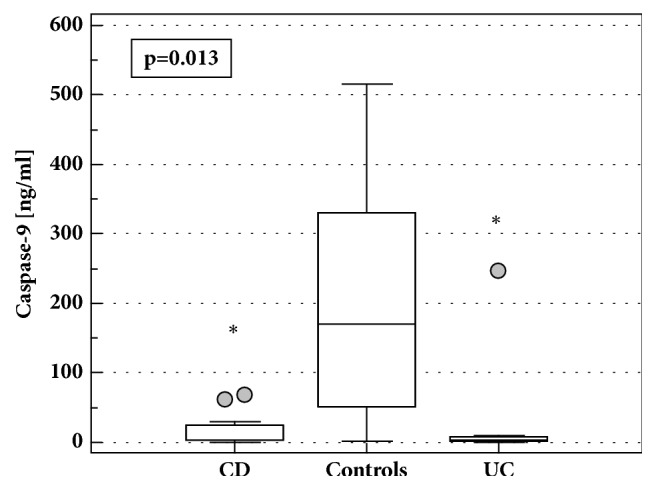
Caspase-9 concentrations in LE-PBMCs lysates from IBD patients and healthy controls. Data presented as medians with 95%CI and analyzed using Kruskal-Wallis* H* test with Conover* post hoc* test. Boxes represent interquartile range, horizontal line – median value, whiskers - 95% CI, and grey dot – far-out values. *∗*, statistically different from controls; CD, Crohn's disease; UC, ulcerative colitis.

The concentrations of caspase 9 were significantly lower in LE-PBMCs from IBD patients than controls (5.12 ng/ml (2.54-10.42)* vs*. 76.3 ng/ml (16.8-346.5),* p*<0.001), regardless the disease type and without significant difference between CD and UC (Figure 4). Caspase 9 concentrations were significantly lower in CD and UC also when differences in protein concentrations between cell lysates were accounted for. Caspase 9 concentration tended to be lower in patients with active IBD (3.51 ng/ml (1.69-7.28)* vs*. 12.85 ng/ml (2.14-77.2),* p*=0.080) and the difference was significant in UC patients (*p*=0.019) but not CD patients (*p*=0.815), also following the adjustment to protein concentrations (*p*=0.023 for UC).

Caspase 9 concentration tended to be lower also in IBD patients treated with AZA (2.98 ng/ml (1.23-7.21)* vs*. 10.9 ng/ml 3.48-34.3),* p*=0.057) or CS (2.46 ng/ml (1.02-5.93)* vs*. 7.96 ng/ml (2.95-21.5),* p*=0.093). With differences in the disease activity as well as protein concentration accounted for, treatment with CS (*p*=0.016) but not AZA (*p*=0.136) had significant impact on caspase 9. Patients without active disease and not treated with CS had the highest caspase 9 concentration (1.6 ng/ml), while these with active disease and treated with CS had the lowest enzyme concentration (0.36 ng/ml).

### 3.5. Interrelationship between Bcl-2, Cytochrome c, p53, and Caspase 9

In a whole cohort, there were positive correlations between Bcl-2 and p53 (*r*=0.56,* p*<0.0001), cytochrome c and p53 (*r*=0.25,* p*=0.049), and caspase 9 and p53 (*r*=0.48,* p*=0.004). Also, Bcl-2 positively correlated with caspase 9 (*r*=0.59,* p*<0.001).

In subgroups, Bcl-2 and p53 were positively correlated in healthy controls (*r*=0.69,* p*<0.001) as well as CD (*r*=0.50,* p*=0.041) but not UC patients. In turn, caspase 9 correlation with p53 and Bcl-2 was significant in UC patients (respectively,* r*=0.69,* p*=0.020 and* r*=0.60,* p*=0.049). In healthy controls, caspase 9 was tightly correlated with Bcl-2 (*r*=0.89,* p*<0.001).

### 3.6. Lymphocyte Count and Bcl-2, Cytochrome c, p53, and Caspase 9

Both absolute and relative lymphocyte counts (part of a peripheral complete blood cell count) were comparable between CD and UC patients (Table 1). Absolute count tended to and relative was significantly lower in active IBD as compared to inactive disease, respectively, 1.76×10^9^/L (1.2-2.2)* vs*. 1.99×10^9^/L (1.6-2.7),* p*=0.100 and 23% (18.1-25.2)* vs*. 29.8% (22.8-33),* p*=0.038.

Treatment with CS was not associated with significant differences in either absolute or relative lymphocyte counts (respectively,* p*=0.156 and* p*=0.686) but IBD patients treated with AZA have significantly lower absolute (1.61 ×10^9^/L (0.9-1.8)* vs*. 2.19×10^9^/L (1.7-2.5),* p*=0.003) and relative (21.2% (14.9-24)* vs*. 28.3% (23-32.9,* p*=0.008) lymphocyte counts.

In a whole cohort, absolute LYM positively correlated with Bcl-2 (*r*=0.35,* p*=0.022), p53 (*r*=0.31,* p*=0.042), and caspase 9 (*r*=0.42,* p*=0.040). Detailed analysis showed absolute LYM to be significantly correlated with Bcl-2 and with caspase 9 exclusively in IBD patients with active disease (respectively,* r*=0.38,* p*=0.040 and* r*=0.53,* p*=0.028) and with p53 exclusively in UC patients (*r*=0.44,* p*=0.030). There was no correlation between LYM and cytochrome c.

### 3.7. Bcl-2, Cytochrome c, p53, and Caspase 9 as IBD and CD Markers

ROC analysis was applied to assess the diagnostic potential of regulators of apoptosis as potential indicators of IBD or CD. Of the evaluated regulators, the markers of IBD, Bcl-2, and caspase 9 were the only ones characterized by good accuracy (Table 2). Combined evaluation of Bcl-2, caspase 9, and cytochrome c increased their accuracy as IBD markers to 92% (95%*CI*: 78-99%). As CD markers, Bcl-2, cytochrome c, and p53 performed significantly better than random marker without discriminating power and all displayed fair accuracy. Of these, logistic regression showed Bcl-2 and cytochrome c to be independently associated with CD presence and their combined evaluation as CD markers increased their accuracy to 85% (74-93%). For comparative purposes, we analyzed the power of hsCRP as CD marker and found it to be unable to differentiate with AUC=0.550, p=0.600.

## 4. Discussion

Our results show significant differences in the concentrations of key players of apoptosis in LE-PBMCs of IBD patients as compared to healthy individuals; however, they do not explicitly indicate the resistance to apoptosis. Under physiological conditions, lymphocytes T in* lamina propria* are more susceptible to apoptosis than circulating lymphocytes, a response to the environment particularly rich in antigens. In IBD, however, they respond aberrantly to proapoptotic signaling and thus contribute to the chronicity of bowel inflammation [22–24]. Expectedly, the abolition of apoptosis resistance forms the basis of many therapeutic strategies applied in IBD which still, despite unquestionable progress, remain unsatisfactory [7, 14, 22, 25]. Owing to its extraintestinal manifestations, IBD is not confined to gastrointestinal tract [16]. And yet, data on peripheral lymphocytes and their sensitivity to apoptosis seems to be scanty and contradictory. On one hand, induction of their apoptosis* via* ECP is viewed as a therapeutic option [15], implying peripheral lymphocytes in IBD to be resistant to apoptosis as well. On the other, however, percentage of apoptotic cells is claimed to be increased in patients with IBD flare and hold responsible for the systemic nature of disease and its extraintestinal manifestations [26]. Although not autoimmune disease* per se*, IBD closely resembles this group of diseases and others, such as multiple sclerosis [27] or type 1 diabetes [28], are associated with impaired apoptotic pathways in peripheral lymphocytes.

There are numerous pro- and antiapoptotic factors and cell fate is decided by the direction in which the balance between them is tipped. Among them, Bcl-2 is a prototypical antiapoptotic molecule and its upregulation seems to be crucial for intestinal lymphocyte resistance to apoptosis. Indeed, intestinal lymphocytes of Crohn's disease patients have been shown to have upregulated expression of antiapoptotic Bcl-2 and Bcl-XL [29] and downregulated expression of proapoptotic Bax [30]. It has also been demonstrated that probiotic bacteria increase the number of apoptotic lymphocytes in the intestinal mucosa by downregulating the expression of Bcl-2. However, counterintuitively and unlike in* lamina propria*, our IBD patients had decreased concentrations of Bcl-2 in LE-PBMCs. Yet, true to its antiapoptotic character, Bcl-2 concentration in LE-PBMCs of our patients was positively correlated with lymphocyte count. Interestingly, when analyzed in subgroups, the correlation between parameters was present in patients with active disease.

Bcl-2 results alone might suggest that LE-PBMCs of IBD patients are more susceptible to apoptosis than these from healthy individuals. Since we found Bcl-2 concentration to drop further in active UC, our results also agree well with these of El-Hodhod et al. [26], who observed the escalation of apoptosis of peripheral lymphocytes in pediatric IBD population. They found the number of both early and late apoptotic cells to increase as compared to healthy children, both in CD and in UC and both in the remission and in active phases of the diseases, although the elevations in UC as well as during the disease flare were significantly higher.

However, similarly to antiapoptotic Bcl-2, the concentrations of other examined molecules, all of a proapoptotic nature, were decreased in IBD as well. Cytochrome c is a key mediator in intrinsic apoptotic pathway, which, upon release from mitochondria, recruits and activates pro-caspases and catalyzes assembly of apoptosomes [31]. Caspase 9, in turn, is a component of apoptosomes and an initiator caspase, catalyzing activation of effector caspase 3 [32]. Consistently, fibroblasts derived from cytochrome c knockout mice [33, 34] are resistant to proapoptotic signals known to trigger intrinsic pathway. Transcription factor p53 regulates the expression of c.a. 500 genes associated with cell response to stress and mutation of its gene,* TP53*, is the most frequent loss-of-function mutation in cancer. Under physiological conditions its concentration is low [35]. Our finding on even lower levels in IBD patients, combined with findings on decreased concentrations of cytochrome c and caspase 9, seems to be strong evidence of apoptosis resistance of LE-PBMCs despite, also decreased, concentrations of Bcl-2. Yet, drawing conclusions is difficult due to the fact that both apoptosis activators and inhibitors as well as caspases play redundant roles and therefore diminished concentrations of one do not necessarily translate into a clear shift of apoptotic balance. Nevertheless, cytochrome c is central to the intrinsic apoptotic pathway and thus, with it being significantly lower, this pathway is likely to be downregulated in LE-PBMCs of IBD patients. Our results, implying intrinsic pathway to be impaired, may still not contradict the observations of El-Hodhod et al. [24]. As reported by Li et al. [31], abrogation of intrinsic pathway yields the cells more susceptible to apoptosis induced by TNF-*α* and involving extrinsic pathway. Concerning divergent results on Bcl-2, it is also worth noting that the correlation pattern of Bcl-2 was counterintuitive to it being an inhibitor of apoptosis. It was positively correlated with proapoptotic cytochrome c, caspase 9, and p53, even though Bcl-2 is known to inhibit apoptosis mediated by p53 [36].

Our findings nevertheless show that, although possibly diminished in IBD in general, intrinsic apoptotic pathway still seems to be upregulated in active inflammation. In line with observations on accelerated apoptosis during IBD flare as compared to remission [26], not only did Bcl-2 drop in active UC, but also cytochrome c increased, significantly so in CD while displaying a similar tendency in UC. Moreover, exclusively in UC, both cytochrome c and p53 concentrations positively correlated with hsCRP, a key marker of systemic inflammation, further corroborating the notion on upregulated intrinsic pathway of apoptosis during inflammation. On the other hand, however, caspase 9 concentration was lower in active UC than in remission.

We analyzed the interrelationship between IBD, apoptosis markers, and lymphocyte count—a part of peripheral complete blood cell count. Similarly to pediatric population [26], there was no difference in absolute and relative lymphocyte counts between our CD and UC patients. Unlike there, however, we found lymphocyte number to be lower in active than inactive IBD, significantly so in case of relative lymphocyte count. There was also positive correlation between lymphocyte counts and Bcl-2, p53, or caspase 9.

Azathioprine is an immunomodulatory drug well established in the treatment of IBD [37]. While mainly acting by blocking* de novo* synthesis of purines and thus DNA replication, it is relatively specific for lymphocytes as these cells lack the salvage pathway of purines synthesis. However, interference with DNA synthesis does not exhaust the immunosuppressive properties of the drug [38]. Among others, azathioprine has been shown to induce the apoptosis in lymphocytes T [39]. Accordingly, also in our cohort, LYM% was lower in patients treated with AZA, agent targeting Bcl-2 mediated apoptosis. There was discrepancy between azathioprine treatments in our patients and when accounted for, we observed that CD patients had lower concentrations of cytochrome c and clearly tended to have lower Bcl-2. Interestingly, in recently published study it was shown that administration of Bcl-2 inhibitor was leading to amelioration of inflammation in experimental model of colitis in mice which deserves attention in searching new therapeutic options [40].

Corticosteroids had opposite effects on leukocyte apoptosis as they have been reported to inhibit apoptosis of eosinophils but stimulate apoptosis of neutrophils [41]. Concerning lymphocytes T, CS are capable of inducing cell shift towards Th2 phenotype and/or trigger apoptosis, depending on their concentration and cellular context [42]. Apoptosis-related genes, the expression of which is directly targeted by CS, remain uncertain but they do not seem to include Bcl-2 [42]. Correspondingly, there was no difference in Bcl-2 levels with respect to CS treatment in our patients. Still, the levels of Bcl-2 are believed to determine the sensitivity of lymphocytes T to proapoptotic signaling conveyed by CS [42]. Therefore, low concentrations of Bcl-2 in peripheral lymphocytes of our IBD patients might be beneficial, implying increased lymphocyte sensitivity to CS. Interestingly, CS treatment was associated with decreased concentrations of caspase 9, independently from lymphocyte count. This effect seems to be counterintuitive; although CS impact directly on caspase 9 expression is unknown, both this caspase and caspase 3, an effector caspase activated by caspase 9, have been implicated in CS-induced apoptosis of lymphocytes T [42]. However, we also found that the disease activity was associated with lower caspase 9 but exclusively in UC patients. We also observed, in multivariate analysis, that the disease activity may influence CS impact on caspase 9 concentration. Therefore, the observation on CS negatively affecting caspase 9 concentrations should be verified on a larger and more homogenate population to separate the effects of CS and the disease activity and phenotype. It would also be of interest to compare caspase 9 concentrations in IBD patients refractory and sensitive to CS treatment.

Considering the disease markers and differential markers are needed to aid in IBD diagnostic process [43], we evaluated the diagnostic power of Bcl-2, cytochrome c, p53, and caspase 9 as biomarkers. Both Bcl-2 and caspase 9 were characterized by good accuracy and cytochrome c was characterized by fair one in discriminating IBD patients from healthy individuals. The diagnostic power could be increased to excellent by simultaneous quantification of Bcl-2, caspase 9, and cytochrome c. Although we had no data on hsCRP in our healthy controls to perform the analysis, literature data, also derived from our group, places hsCRP accuracy at 83-90% [44–47]. Therefore, together, apoptosis-associated markers quantified in lymphocytes seem to display superior accuracy to hsCRP, key marker of systemic inflammation [48] and one of a few biochemical markers of clinical relevance in IBD [43]. However, this finding has to be validated on larger cohort and with apoptosis-associated markers assessed simultaneously with hsCRP. As the concentrations of Bcl-2, cytochrome c, and p53 were lower in CD than UC, these were evaluated as potential CD markers. The accuracy of all these markers in discerning CD patients was significantly better than that of a chance marker and their performance fair. By comparison, hsCRP had no power in either present cohort or others reported [44]. The model of logistic regression was built on Bcl-2 and cytochrome c as the only significant contributors. The accuracy of combined quantification of Bcl-2 and cytochrome c was improved as compared to the quantification of individual markers and reached the threshold of 85%, a lower limit for biomarkers used in clinical practice. Taking into consideration the similarity of CD and UC symptoms, our finding is of utmost importance and clinical relevance. It is frequently difficult to distinguish CD of the colon-rectum from UC with no golden standards for differential diagnosis available. Consequently, it is estimated that proper diagnosis may elude up to 10% of IBD patients. Inaccurate diagnosis affects the disease management and patients' prognosis. It is also associated with increased risk for patients and cost for health care system, resulting from repeated testing required for diagnosis reevaluation [49].

Our study presents preliminary results and has several limitations that ought to be stressed. Firstly, it was conducted on relatively small group of patients but testing for required sample size showed it to be adequate for the key results (differences in concentration between healthy individuals and CD and UC patients as well as the results of ROC analysis) to have sufficient statistical power. Secondly, for economic reasons, data on lymphocyte counts or hsCRP were unavailable for apparently healthy individuals to allow for more thorough analysis. Thirdly, our patients were not naive but the issue was addressed by conducting treatment-adjusted analysis if appropriate. Fourthly, caspase 9 assays employ monoclonal antibodies detecting active form of caspase but when inquired, the assay manufacturer did not exclude the possibility of them reacting with an inactive enzyme.

Concluding, although not completely unambiguously, our results imply diminished apoptotic capacity of LE-PBMCs in IBD in terms of its intrinsic pathway but, simultaneously, an upregulation of proapoptotic features parallel to increasing severity of systemic inflammation represented by hsCRP. Observed abnormalities in the concentrations of key players in intrinsic pathway of apoptosis seem to be more pronounced in CD. Upon positive validation on a larger set of patients, combined quantification of Bcl-2 and cytochrome c might be considered as an adjunct in differential diagnosis of UC and CD of colon and rectum.

## Figures and Tables

**Table 1:** Demographic and clinical characteristics of study participants.

	**Crohn's disease**	**Ulcerative colitis**	**Controls**	***P* value**
**Number of observations**	17	25	22	
**Anthropometric data:**				
** Female/male, n**	19/8	12/13	14/8	0.553^*χ*2^
** Age, median [yrs.]**	34 (30-36)	34 (26.3-51)	40.5 (30-50)	0.370 ^K^
** BMI, median [kg/m** ^**2**^ **]**	21.7 (18.6-24.5)	22.5 (19.5-24.9)	24.1 (19.6-29.8)	0.457 ^K^
**Disease activity:**				
** Active IBD, n (**%**)**	13 (76.5%)	17 (68%)		0.731 ^F^
** CDAI/MDAI** ^**1**^	192 (145-329)	5 (2-6)	-	
**Blood parameters:**				
** ESR, median [mm/h]**	18 (12-28)	18 (10-36)	-	0.720 ^M^
** hsCRP, median [mg/L]**	9.4 (5.7-26.8)	3.6 (4.9-20.2)	-	0.603 ^M^
** HGB, mean [g/dL]**	12.2 (11.1-13.3)	11 (9.9-12)	-	0.085 ^t^
** WBC, median [×10** ^**9**^ **/L]**	7.86 (6.77-8.95)	8.99 (7.43-10.54)	-	0.223 ^W^
** PLT, mean [×10** ^**9**^ **/L]**	320 (261-378)	374 (319-428)	-	0.169 ^t^
** LYM, mean [**%**]**	24.5 (18.9-30)	23.5 (19-28.1)	-	0.789 ^t^
** LYM, mean [×10** ^**9**^ **/L]**	1.98 (1.27-1.68)	1.97 (1.47-2.48)	-	0.991 ^t^
**Treatment:**				
** ASA, n (**%**)**	16 (94.1%)	22 (88%)		0.636 ^F^
** CS, n (**%**)**	5 (29.4%)	14 (56%)		0.120 ^F^
** AZA, n (**%**)**	0 (0%)	22 (100%)		<0.001 ^F^
**Apoptosis:**				
** Bcl2, median [ng/ml]**	15.04 (11.4-20)^2^	19.52 (13.5-23)^2^	37.6 (32.8-48.8)^3,4^	<0.00001 ^K^
** cyt. c, median [ng/ml]**	235 (97.4-329)^2^	326 (271-358)^2^	406 (286-434)^3,4^	0.003 ^K^
** p53, median [ng/ml]**	3.07 (2.58-6.56)^2^	8.5 (3.32-11.1)	15.5 (4.58-18.4)^3^	0.038 ^K^
** casp. 9, median [ng/ml]**	3.54 (2.96-26.7)^2^	3.43 (1.26-8.64)^2^	170 (25.9-350)^3,4^	0.013 ^K^

If not otherwise stated, data presented as means or medians with 95%*CI*.

n, number of observations; ESR, erythrocyte sedimentation rate; ^*χ*2^, Chi-square test;  ^M^, Mann-Whitney *U* test; K, Kruskal-Wallis *H* test; F, Fisher exact test; W, Welch test; t, t test for independent samples; ASA, 5′-aminosalicylate (5′-ASA) derivatives; CS, corticosteroids; AZA, azathioprine; cyt., cytochrome; casp., caspase; ^1^, Disease activity presented as median CDAI score for Crohn's disease and median MDAI score for ulcerative colitis; ^2^, significantly different from controls; ^3^, significantly different from CD; ^4^, significantly different from UC.

**Table 2:** Discriminative power of apoptosis regulators as IBD and CD markers.

	**IBD marker **(CD+UC *vs*. controls)			
	**Bcl-2**	**cytochrome c**	**p53**	**caspase 9**

**AUC (95**%***CI*), p**_(AUC=0.5)_	0.86 (0.75-0.93), <0.0001	0.73 (0.6-0.83), <0.001	0.63 (0.5-0.75), 0.107	0.82 (0.65-0.93), 0.002
**Sens. and spec.**	85.7 and 81.8%	90.5 and 54.5%	88.1 and 54.5%	87.5 and 80%
**Criterion [ng/ml]**	≤25	≤390.4	≤15.2	≤29.9

	**CD marker **(CD *vs*. controls+UC)			
	**Bcl-2**	**cytochrome c**	**p53**	**caspase 9**

**AUC (95**%**CI), p**_(AUC=0.5)_	0.78 (0.66-0.88),<0.0001	0.73 (0.61-0.84), 0.002	0.7 (0.57-0.81), 0.003	na
**Sens. and spec.**	94.1 and 59.6%	64.7 and 78.7%	100 and 38.3%	na
**Criterion [ng/ml]**	≤22.8	≤242	≤14.8	na

IBD, inflammatory bowel disease; CD, Crohn's disease; UC, ulcerative colitis; AUC, area under receiver operating characteristics (ROC) curve; Sens. and spec., sensitivity and specificity; na, non-applicable.

## Data Availability

The data used to support the findings of this study are available from the corresponding author upon request.
